# A poor man’s coherent Ising machine based on opto-electronic feedback systems for solving optimization problems

**DOI:** 10.1038/s41467-019-11484-3

**Published:** 2019-08-08

**Authors:** Fabian Böhm, Guy Verschaffelt, Guy Van der Sande

**Affiliations:** Applied Physics Research Group, Vrije Universiteit Brussel, Pleinlaan 2, 1050 Brussels, Belgium

**Keywords:** Optoelectronic devices and components, Information theory and computation, Complex networks

## Abstract

Coherent Ising machines (CIMs) constitute a promising approach to solve computationally hard optimization problems by mapping them to ground state searches of the Ising model and implementing them with optical artificial spin-networks. However, while CIMs promise speed-ups over conventional digital computers, they are still challenging to build and operate. Here, we propose and test a concept for a fully programmable CIM, which is based on opto-electronic oscillators subjected to self-feedback. Contrary to current CIM designs, the artificial spins are generated in a feedback induced bifurcation and encoded in the intensity of coherent states. This removes the necessity for nonlinear optical processes or large external cavities and offers significant advantages regarding stability, size and cost. We demonstrate a compact setup for solving MAXCUT optimization problems on regular and frustrated graphs with 100 spins and can report similar or better performance compared to CIMs based on degenerate optical parametric oscillators.

## Abstract (web summary)

Coherent Ising machines are an unconventional optical computing architecture for solving optimization problems. Böhm et al. present a design based on opto-electronic feedback systems that avoids using nonlinear optical materials, improving the prospects for scalability and reliable operation.

## Introduction

Recent advances in optical and quantum computing are paving the way for new computational paradigms, which may soon replace conventional digital computers in challenging tasks. Of particular interest are combinatorial optimization problems, which are often known to be NP-hard and thus considered hard to solve efficiently on digital computers^1^. To speed up calculation time compared to digital hardware, different non-von Neumann architectures have been proposed that attempt to solve optimization problems by mapping them to Ising models. Finding the optimal solution then becomes equivalent to finding the ground state of the Ising model^2^, which is implemented with networks of coupled artificial Ising spins that can be realized with various physical systems, e.g. Josephson junctions, trapped ions, or optical states^3–9^. The energy function of these so-called Ising machines is proportional to the Ising Hamiltonian, so that they will naturally evolve to the ground state of the Ising model and thus to the optimal solution. As the evolution to the ground state typically occurs on very fast timescales, Ising machines promise a considerable speed up over conventional algorithms in finding solutions to optimization problems^10,11^, which will have significant implications for various important areas such as finance, pharmaceutics, logistics, or machine learning.

Among the different concepts, coherent Ising machines (CIMs) have attracted recent interest^12^. CIMs use the formal similarities between the Ising Hamiltonian and the Hamiltonian of bistable interfering coherent optical states to realize large-scale Ising machines with networks of coupled optical states. This offers a number of advantages over other Ising machines such as quantum annealing, as CIMs are able to operate at room temperature, can be constructed from off-the-shelf photonic components, and are capable of implementing arbitrary coupling topologies^12^. CIMs are also gain-dissipative systems, which makes them efficient in escaping local energy minima and thus suitable for solving optimization problems^13^. The optimal solution then represents the lowest loss configuration and can be found by driving the system close to the minimum gain threshold, where other local energy minima are not stable yet. Based on this, various types of CIMs have been proposed that implement Ising spin networks with bistable coherent optical states, such as coupled lasers^7,14^ and degenerate optical parametric oscillators (DOPOs)^8^. Current state-of-the-art CIMs based on DOPOs have demonstrated their ability as global optimizers for various large-scale problems^11,15–17^. By taking advantage of the large bandwidth of optical systems, they can operate at high speed and have shown speed-ups over conventional algorithms^11,18^.

However, the generation, interference and detection of the coherent optical states is phase-sensitive and thus makes stable operation technically challenging. In DOPO-based CIMs, artificial Ising spins are represented by the optical phase of short laser pulses that are generated by nonlinear optical processes and circulated inside a 1 km long ring fiber cavity^8^. This requires phase-stability for the whole length of the cavity and makes the system highly susceptible to external perturbations, often leading to cases where unstable conditions deteriorate performance^16,19^. Furthermore, the nonlinear DOPO generation process demands powerful laser systems and temperature-controlled nonlinear materials, which results in large and complex optical setups. These drawbacks make CIMs challenging to build and operate and hinder realization as small and cost efficient devices, e.g. as photonic integrated circuits.

Here, to improve the stability and decrease the footprint of CIMs, we introduce a different concept by implementing a CIM based on opto-electronic oscillators (OEOs) subjected to self-feedback. OEOs are an attractive choice since they can easily be built from few off-the shelf components or as photonic integrated circuits and are known for their inherent stability and complex nonlinear dynamics, which are used in various applications, e.g. cryptography, microwave generation, and optical neuronal computing^20^. We demonstrate how the rich bifurcation structure of OEOs can be used to generate arbitrarily large controllable artificial spin networks. Our concept results in a more compact experimental setup, which requires only a few components. We test its performance in solving optimization problems with up to 100 spins and find that it is suitable as a solver for MAXCUT optimization problems with a similar or better performance compared to DOPO-based CIMs. Contrary to DOPO-based CIMs, our machine does not require external cavities or nonlinear optical processes, which drastically decreases its cost and its footprint, while also enhancing its stability. This demonstrates the large potential of feedback systems in general to be used for computation of the Ising model.

## Results

### Implementing artificial Ising spin network with OEOs

The Ising model describes an ensemble of binary spins *σ*_*n*_, which are either in the spin up *σ*_*n*_ = 1 or the spin down state *σ*_*n*_ = −1. Interaction of the spins is achieved by coupling them using the spin coupling topology *J*_*mn*_. The energy function of an ensemble of *N* coupled spins is then given by the Ising Hamiltonian

1 $$H_{{\mathrm{Ising}}} = - \frac{1}{2}\mathop {\sum}\limits_{mn}^N {J_{mn}} \sigma _m\sigma _n.$$

**Fig. 1:**
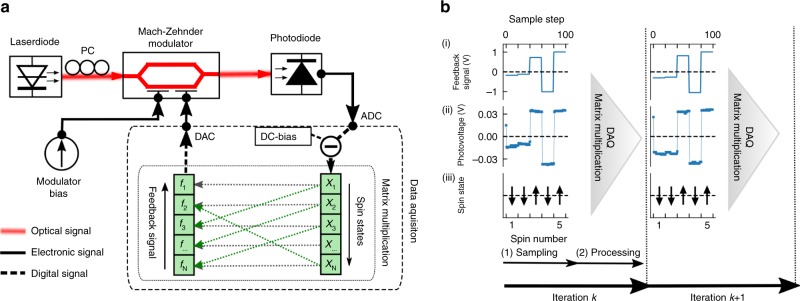
Experimental schematic and working principle. **a** Experimental schematic of an OEO-based coherent Ising machine. PC polarization controller, ADC analog–digital converter, DAC digital–analog converter. **b** Working principle during two consecutive iterations. In the sampling stage (1), the feedback signal is generated (i) and the resulting spin amplitude is measured (ii). The spins states are then determined by the sign of the spin amplitude (iii). In the processing stage (2), spins are digitally processed and the matrix multiplication of Eq. (3) is performed

As shown in ref. ^21^, the Hamiltonian of interfering optical coherent states is analogous to the Ising model, where the spin coupling *J*_*mn*_ corresponds to the optical phase difference Δ*φ* and the optical coupling strength of the interfering states. For example, the Ising Hamiltonian corresponds to coupled DOPOs^21^, where the phase difference is fixed to Δ*φ* = {0, *π*}. Due to this phase degeneracy, the electrical field is real valued with a positive or negative amplitude. Motivated by this, we suggest to remove the phase sensitivity in CIMs with an OEO, which consists of an optical and an electrical pathway (see Fig. 1a). The optical pathway implements a nonlinearity by feeding the output of a laser diode through a Mach–Zehnder modulator (MZM) and detecting it with a photodiode. The electrical pathway creates time-discrete feedback by sampling the photovoltage and feeding it back to the input of the MZM. Inside the MZM, the coherent input is split and interfered with itself. A phase difference corresponding to the feedback signal is set within one of the arms by a phase modulator and the output of the MZM is then the squared in-phase component of the interfering electrical fields. In the Methods section, we show how this output approximates the coherent superposition of DOPO pulses in DOPO-based CIMs. By coupling the output of multiple OEOs together, either electrically or optically^22,23^, a network of bistable optical states can thus be generated to represent an ensemble of Ising spins. Contrary to DOPO-based CIMs however, generation, interference, and detection of the optical states is fully contained within the MZM and the feedback system. All information about the optical states is encoded in the light intensity outside of the MZM and therefore phase sensitivity is removed.

To map the OEO network to a network of Ising spins, we exploit the nonlinear nature of the opto-electronic feedback system. For an ensemble of coupled OEOs with time-discrete feedback, the time evolution of the *n*th OEO *x*_*n*_[*k*] during iteration step *k* is given by the following nonlinear map^23^:

2 $$x_n[k + 1] = {\mathrm{cos}}^2\left( {f_n[k] - \pi /4 + \zeta _n[k]} \right) - \frac{1}{2}.$$

Gaussian white noise *ζ*_*n*_[*k*] and a constant bias of −*π*/4 are applied to the system. The feedback term *f*_*n*_[*k*] is calculated according to

3 $$f_n[k] = \alpha x_n[k] + \beta \mathop {\sum}\limits_m {J_{mn}} x_m[k].$$

*f*_*n*_[*k*] includes both self-feedback to each oscillator *x*_*n*_[*k*] with the feedback strength *α* as well as mutual coupling with the coupling matrix *J*_*mn*_ and the coupling strength *β*. Without mutual coupling (*β* = 0), it can easily be shown by linear stability analysis that the system undergoes a pitchfork bifurcation at *α* = 1. Below the bifurcation point, the system has only one stable fixed point $x_1^ \ast = 0$, while above the bifurcation point the system has two stable fixed points $x_{2,3}^ \ast = \left\{ { - a_0,a_0} \right\}$ and one unstable fixed point $x_1^ \ast = 0$. The pitchfork bifurcation results in a symmetrical bistability, where there is an equal probability that a single oscillator will end up in either of the two stable fixed points when the system is initially in the unstable fixed point. Ising spin networks are then generated by mapping the photovoltage *x*_*n*_[*k*] to the Ising spins *σ*_*n*_ by *σ*_*n*_ = *sig*_*n*_(*x*_*n*_[*k*]). As DOPO-based CIMs have to implement this kind of bistability with a phase-sensitive nonlinear optical amplification process, using the nonlinearity of an MZM subjected to self-feedback presents a significantly easier and more stable approach to realizing large-scale spin networks.

To facilitate the realization and coupling of several OEOs, we employ a time-multiplexing scheme that allows us to emulate a large ensemble of oscillators with a single system. For a network of *N* spins, the feedback signal is divided into *N* equal intervals, where each individual interval represents a single artificial spin (see Fig. 1b). The feedback signal and the photovoltage are then represented by piecewise constant functions and are generated and read out sequentially. Similar to DOPO-based CIMs, we employ a hybrid computing scheme where multiplexing and coupling are performed by digital hardware while the nonlinear system is implemented with the optical system. For each iteration, the photovoltage *x*_*n*_ and the feedback signal *f*_*n*_ for each spin *n* are updated in a two-stage process, namely a sampling and a processing stage (see Fig. 1b). In the sampling stage, the multiplexed feedback signal is injected from a digital–analog converter (DAC) to the MZM and the resulting photovoltage is sampled by an analog–digital converter (ADC). In the processing stage, the signal is demultiplexed, a matrix multiplication is performed to facilitate the spin coupling and the resulting feedback signal is multiplexed again for the next iteration. This hybrid computing scheme presents a good compromise as it allows to implement arbitrary networks while taking advantage of some of the high bandwidth of the optical system. In DOPO-based CIMs, this enables fast computation times at rates of hundreds of megahertz per spin, which can already outperform other heuristic methods^11,18^. However, an all-optical approach could remove any slowdown of the digital I/O system and take full advantage of the optical system to further increase the rate to tens of gigahertz.

**Fig. 2:**
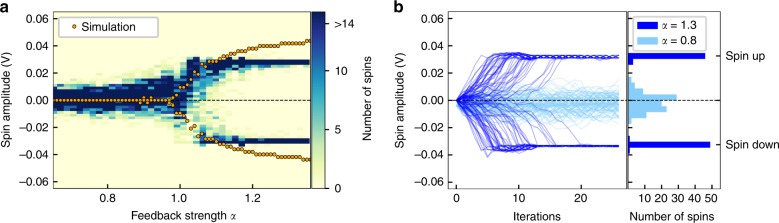
Behavior of uncoupled artificial spins at the pitchfork bifurcation. **a** Distribution of the spin amplitude for 100 uncoupled artificial Ising spins after 100 iterations as the feedback strength *α* is varied. At *α* = 1, the system undergoes a pitchfork bifurcation. The data are overlaid with simulations of Eq. (2) as orange dots. **b** Time evolution of spin amplitude for feedback strengths of *α* = 0.8 (turquoise) and *α* = 1.3 (blue) and the respective distribution of amplitudes

We test the behavior of the OEO-based CIM as an artificial spin network by emulating an ensemble of 100 uncoupled spins (*J*_*mn*_ = 0). Figure 2a shows the amplitude distribution for all spins after 50 iterations as we increase the feedback strength *α* from below to above the bifurcation point. Below the bifurcation point, only the trivial fixed point $x_1^ \ast = 0$ is stable and the artificial spins are distributed around $x_1^ \ast$. As the feedback strength increases above *α* = 1, the trivial fixed point becomes unstable and the artificial spins bifurcate into the two new stable fixed points $x_{2,3}^ \ast = \pm a_0$. As expected for a pitchfork bifurcation, the amplitude *a*_0_ of the stable fixed points increases with the feedback strength, which also agrees well with simulations of the nonlinear map (2) (orange dots in Fig. 2a). For higher feedback strengths, we observe a deviation from the nonlinear map (2) as the spin amplitude starts to saturate due to load limitations of the DAC. Figure 2b shows two exemplary time evolutions for all spins below (*α* = 0.8) and above (*α* = 1.3) the bifurcation point. At *α* = 0.8, we observe how the spins are fluctuating around the stable fixed point $x_1^ \ast$ as a consequence of the system’s noise. At *α* = 1.3, the noise drives the spins away from the now unstable fixed point $x_1^ \ast$ as the spin amplitude bifurcates into the new stable fixed points $x_{2,3}^ \ast$. From the histogram, we see that 49 spins are in the spin up state and 51 spins are in the spin down state, which exemplifies that there is an equal probability for both configurations. For 50 independent measurements, we find that the average probability for the spin up and spin down configuration are *P*_up_ = 0.49 ± 0.08 and *P*_down_ = 0.51 ± 0.08, thereby corroborating that the artificial spins are emulating the correct behavior of independent Ising spins.

### Solving MAXCUT optimization problems with OEO-based CIMs

To demonstrate the capability of OEO-based CIMs as general solvers for optimization problems, we perform different benchmarks for the MAXCUT problem. MAXCUT is the task of dividing a graph *J*_*mn*_ into two subsets with a maximal number of connecting edges between both subsets and is known to be an NP-hard problem^1^. MAXCUT problems can easily be transformed to an Ising model by implementing the graph structure *J*_*mn*_ and setting each non-zero edge to be antiferromagnetic (*J*_*mn*_ = −1). The energy minimum of the Ising Hamiltonian is then equivalent to the maximal cut^11^. In the following, we perform ground state searches for MAXCUT problems in various graph structures to demonstrate that OEO-based CIMs can find the correct solution. As a first instance, we consider a square lattice, where the spins are organized in a two-dimensional (2D) grid structure and coupled to their four nearest neighbors. The ground state is given by a checkerboard pattern, where neighboring spins are alternating between the spin up and down configuration.

**Fig. 3:**
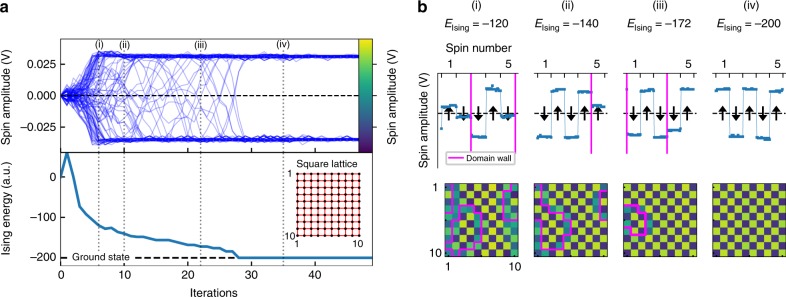
Spin dynamics during a ground state search in a square lattice. **a** Time evolution of spin amplitude and Ising energy during the ground state search in a 10 by 10 square lattice for *α* = 0.25, *β* = 0.29. (inset) Graph structure of the square lattice. Black dots indicate the position of spins and red links indicate antiferromagnetic coupling. Links for periodic boundary conditions are not visualized. **b** Snapshots of the artificial spins during the ground state evolution. The spin amplitude is color coded, so that yellow indicates spin up and blue indicates spin down. The respective color bar is shown in **a**. Domain walls are indicated by magenta lines. Also shown is the sampled photovoltage signal and the respective spin state for the first 5 spins in row 5

We implement a 10 by 10 square lattice with periodic boundary conditions and study the ground state evolution. In Fig. 3a, we show the time evolution of each spin’s amplitude and the corresponding Ising energy. Starting from the unstable fixed point $x_1^ \ast$, the system is driven across the threshold of the pitchfork bifurcation where the spins start to bifurcate into the two stable fixed points $x_{2,3}^ \ast$. The mutual coupling drives the spins to reorder themselves, which is accompanied by a drop in the Ising energy. This process continues until after 28 iterations, the ground state energy has been reached. To better understand the evolution to the ground state, we show snapshots ((i)–(iv) in Fig. 3b) of the spin amplitude during the ground state search. The spins are color coded, so that yellow indicates spin up and blue indicates spin down. We find that, as the artificial spins start to bifurcate, they organize themselves into small circular domains ((i) in Fig. 3b). Within these domains, spins are already organized in a checkerboard pattern. At the boundaries, domain walls are formed, which create a mismatch in the pattern (indicated by magenta lines). As the ground state search progresses, the domain walls start to move and the domains merge and shrink ((ii)–(iii) in Fig. 3b) until all of them eventually vanish and the ground state is reached ((iv) in Fig. 3b). It is interesting to note here that these domain evaporation dynamics are likewise observed in DOPO-based CIMs^17,24^.

### MAXCUT performance in regular and frustrated graphs

**Fig. 4:**
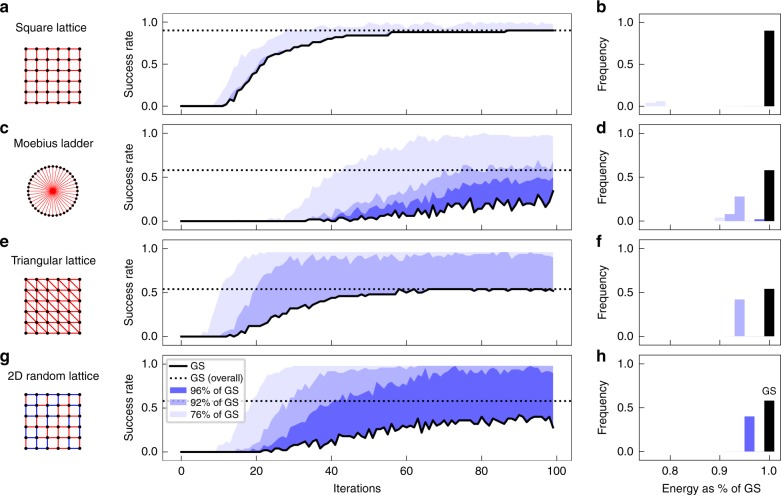
Time evolution of success rate and energy distribution for various frustrated graphs. Time evolution of the success rate in 50 independent trials to reach the ground state (black line) and 96%, 92%, and 72% of the ground state energy (indicated by shades of blue) as well as the respective histogram of the minimum energies for various graphs with *N* = 100 spins. Investigated graphs are the square lattice (**a**) and (**b**) (parameters used: *α* = 0.25, *β* = 0.29), the Möbius ladder graph (**c**) and (**d**) (*α* = 0.07, *β* = 0.39), the triangular lattice (**e**) and (**f**) (*α* = 0.32, *β* = 0.57), and a 2D random lattice (**g**) and (**h**) (*α* = 0.18, *β* = 0.32). The sketches show the respective coupling scheme, where red connections indicate antiferromagnetic links (*J*_*mn*_ = −1) and blue connections indicate ferromagnetic interactions (*J*_*mn*_ = 1)

To assess the performance of the OEO-based CIM, we evaluate the success rate to reach the ground state in 50 independent experiments. This indicates whether it is likely that the machine becomes trapped in local energy minima. In Fig. 4a, we show the time evolution of the success rate for the square lattice. After around 100 iterations, 90% of the instances are able to reach the ground state, which indicates a high probability to escape local minima. Only 10% of the calculations get stuck in local energy minima, so that all instances are within 76% of the ground state energy (indicated by shades of blue in Fig. 4a and the histogram in Fig. 4b). While the square lattice can be considered as an easy problem that can be efficiently solved by computer algorithms^25^, we also evaluate the performance in frustrated lattice structures, where competing spin interactions lead to a disordering and results in more complex ground state patterns. Typically, frustrated lattices can be more challenging to solve due to local energy minima and critical slowing down and thus present a challenge to conventional solvers such as Monte Carlo methods and Hopfield networks^26,27^. In the following, we consider the Möbius ladder graph, which is a cubic ring graph, where each spin is also connected to its neighbor on the opposite site of the ring. When *N*/2 is an even number for *N* spins, competing spin interactions due to the antiferromagnetic coupling to neighboring spins cause lattice frustrations. In Fig. 4c, we show the time evolution of the success rate for a Möbius ladder graph with 100 spins. After 100 iterations, the CIM achieves a success rate of 34%. From the timeseries, we can also observe that the system can escape from the global energy minimum, so that the overall success rate is higher when we consider the total number of instances that have reached the ground state at some point. In this case, the CIM achieves an overall success rate of 59% (indicated by the dotted line in Fig. 4), which is almost three times higher than previously reported values for DOPO-based CIMs^15^. For the distribution of energies, we find that all unsuccessful calculations lie within 90% of the correct ground state energy, which presents a good approximation of the global minimum (see Fig. 4d). Similar or enhanced performance can also be observed for other frustrated graphs. In Fig. 4e and g, we test the success rate to reach the ground state for the triangular lattice and a 2D random lattice. In the triangular lattice, frustrations are caused by coupling of each spin to its six nearest neighbors. This results in various degenerate ground states, which for example relate to the ground state in quantum spin liquids in YbZnGaO_4_ (ref. ^28^). For a 10 by 10 triangular lattice, the CIM achieves a success rate of 52% after 100 iterations, while almost all other instances are a good approximation at 94% of the ground state energy (see Fig. 4f). In a 2D random lattice, frustrations are caused by randomly choosing between ferromagnetic and antiferromagnetic coupling for each edge. We evaluate the success rate for a randomly generated graph and find that the CIM achieves an overall success rate of 58%. This is an improvement when compared to the success rate reported for DOPO-based CIMs, where an average of 28% was reported for various 2D random graphs^17^. The remaining states all fall within 96% of the correct solution and thus again present a good approximation (see Fig. 4h).

**Fig. 5:**
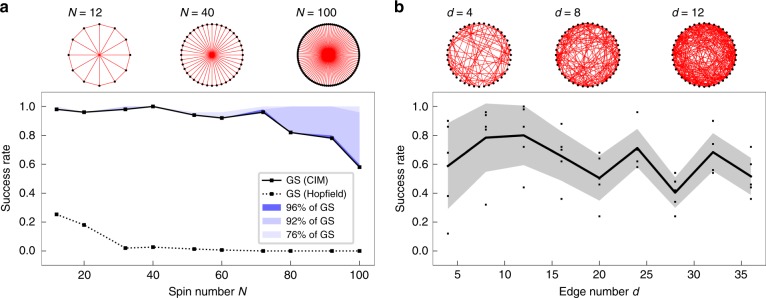
Scaling of the success rate with graph size and edge density. **a** Scaling of the overall success rate with the graph size for the Möbius ladder graph after 100 iterations. The data are compared to the success rate to reach the ground state with a Hopfield network (dashed line). **b** Scaling of the average success rate with the number of edges per node for five different random graphs with *N* = 40 spins after 200 iterations. The gray area shows the standard deviation from the average

We also investigate how the graph properties influence the success rate of the CIM. For the Moebius ladder graph, we consider the scaling of the overall success rate with the graph size *N* in Fig. 5a. As the graph size decreases, the success rate quickly increases to around 100% for *N* ≤ 72 spins. This overall success rate is higher when compared to DOPO-based CIMs, where a 100% success rate for the Möbius ladder graphs was reported only for *N* ≤ 16 spins^15^. This advantage becomes more pronounced in comparison to classical Hopfield networks, which we have implemented with the Metropolis–Hastings algorithm at low temperatures. In that case, for the smallest graph *N* = 16, only 34% of 50 independent instances reach the ground state. As the graph size is increased, the success rate for the Hopfield network quickly drops to zero so that no instance is able to find the correct ground state for *N* = 100. This demonstrates that the CIM can be more efficient in escaping local energy minima. This is also corroborated by the energy histograms in Fig. 4, where all calculations end up in configurations at or very close to the global minimum.

Another important measure which influences the success rate is the density of edges in a graph. For quantum annealing for example, the embedding of dense or complete graphs can be challenging so that the success rate is poor compared to CIMs or classical algorithms^16^. Here, we consider sparse and dense random graphs, where each node has the same number of randomly distributed links. In Fig. 5b, we show the average success rate for five random graphs with *N* = 40 spins after 200 iterations as the edge density *d* is increased from a sparse connectivity to an almost complete graph. We find that the success rate is mostly unaffected by the edge density and reaches over 50% in most instances, which is also comparable to previous results in DOPO-based CIMs and significantly more successful than quantum annealing based machines^16^. It has to be stressed here that the DOPO-based CIM applies an annealing scheme in ref. ^16^, where the system is slowly driven above the gain threshold with a linear annealing schedule. While we observe similar performance without the need for annealing, we expect that the success rate could be further increased at the cost of longer calculation times by adapting the same annealing technique. Overall, our benchmarks demonstrate that the OEO-based CIM is capable of finding the optimal solution for various difficult optimization problems and achieves similar or even better performance than current state-of-the-art CIMs.

## Discussion

We have proposed and implemented a CIM, which is built from a compact opto-electronic feedback system. Compared to quantum annealing devices, our machine benefits from the same advantages as DOPO-based CIMs, while having a more compact and more controllable setup. OEO-based CIMs do not require an external ring fiber cavity and nonlinear optical processes and the overall footprint and cost can be significantly decreased. As OEOs can be fabricated as photonic integrated circuits^29,30^, they will enable miniaturization of cheap, fast and fully programmable CIMs. OEO-based CIMs also provide a significant enhancement in regard to the overall stability of CIMs. While the phase-sensitive setup of DOPO-based CIMs can deteriorate performance and requires filtering of the results^16^, the OEO-based machine encodes spins in the light intensity. As a consequence, we can report stable conditions over several hours of operation without the need for active stabilization or additional filtering of results. This increased stability allows to operate the CIM closer to the gain threshold, which results in high success rates. It also opens up CIMs to other fields of study, for example the statistical properties of artificial spin networks with strong injected noise.

The performance of OEO-based CIMs as a general solver for optimization problems was demonstrated by solving various difficult MAXCUT problems. We find that the machine is able to find the correct ground state for all graphs, even in the presence of lattice frustrations where simple Hopfield networks are stuck in local energy minima. Compared to DOPO-based machines, we can report similar or increased performance in the success rate for all graphs. Similar to DOPO-based CIMs, we expect that the overall success rate can be further increased by gradually increasing the feedback and the coupling strength^16^, which suggests that similar good performance can be expected for various tasks. In terms of computational speed, our system is interesting from different perspectives. On the one hand, as the dynamical equations are significantly simpler compared to those of other CIM systems^7,21^, they are considerably more efficient to simulate on digital computers and provide a fast general purpose method for solving optimization problems on conventional hardware. On the other hand, the high bandwidth of the optical system allows for computation speeds that can potentially surpass conventional methods. While fast calculation times per iteration are not the focus of our current setup, the compact optical system is designed to be compatible with the fast field programmable gated array (FPGA)-based measurement-feedback systems of DOPO-based CIMs. By using an FPGA system, ground state searches within only a few milliseconds are possible in the future^11,15^ and further improvements could be achieved by using faster opto-electronic components or sampling from several OEOs in parallel.

Due to the general nature of our proposed system, our work shows that it should be possible to realize similar CIMs with other nonlinear feedback systems. Since the cos^2^ nonlinearity can be approximated with various functions, a realization with analog electronics for example could be easy to implement and provide similar speed. Nonlinear feedback systems as a whole thus become interesting for the study of optimization problems and will be an intriguing alternative to other artificial Ising spin systems. However, the advantages of using an optical feedback system to implement Ising spin networks will prevail in an all-optical setup. Since OEO-based CIMs can be implemented as integrated photonic circuits, it will be possible to combine them with the recent development of programmable silicon photonic circuits to realize flexible spin coupling all optically^31^. This will enable building programmable and energy efficient CIMs as a single photonic integrated circuit and remove the digital I/O system altogether, hence taking full advantage of the high bandwidth of the optical system and yielding a significant speed up over existing CIM concepts. Combining a cheap and compact setup with the performance of DOPO-based CIMs, OEO-based CIMs thus make CIMs more accessible and promise to overcome important hurdles towards their practical application.

## Methods

### OEO-based CIM

For the OEO-based CIM, we employ a single time-multiplexed feedback loop, which was similarly employed to study synchronization phenomena in arbitrary networks^23^. The coherent signal is emitted from a single-mode wavelength-stabilized DFB laser diode at a wavelength of *λ* = 1.55 μm. The laser is operated at around two times its threshold current at an optical power of 0.3 mW. The optical signal is transmitted through single-mode optical fibers. To reduce the negative effects of optical feedback to the laser, all optical fibers use angled connectors and an additional optical isolator is inserted directly behind the laser. We use a 13 GHz Lithium Niobate MZM to modulate the optical signal. The angle of polarization for the laser light is adjusted by a polarization controller in front of the modulator. To shift the bias phase Δ*φ* of the modulator, a constant DC bias of $V_{{\mathrm{bias}}} = \frac{1}{4}V_\pi$ is applied. It has to be noted here that this results in a positive bias of ${\mathrm{\Delta }}\varphi = \frac{\pi }{4}$ instead of a negative bias as in Eq. (2). The cos^2^ is symmetric under change of sign (cos^2^(Δ*φ*) = cos^2^(−Δ*φ*)); however, since the slope of cos^2^ is antisymmetric (−2sin(Δ*φ*) = 2sin(−Δ*φ*)), the sign of the feedback strength *α* and the coupling strength *β* has to be inverted to implement the correct spin interaction. To relate the coupling and feedback strength in the experiment to *α* and *β*, we rescale the voltage signals with the voltage at the bifurcation point obtained in Fig. 2a.

For the signal generation and subsequent sampling, we use an USB data acquisition interface which is coupled to a computer. In the sampling stage, the feedback signal is generated by a 14-bit DAC from the stored values of the previous iteration at a sampling rate of 6000 Sa/s. The optical signal from the MZM is detected and transformed to an electrical signal by a photodiode with a 150 MHz bandwidth and sampled at 6000 Sa/s by a 14-bit ADC. To ensure synchronous generation and sampling, the data acquisition is triggered by an external signal generator operating at 1 Hz. This results in a computation time of 1 s per iteration. We want to remark that this long computation is primarily due to the data acquisition interface and can be performed much faster with more sophisticated hardware.

During the data acquisition, a network of *N* spin is generated by time-multiplexing, where the acquisition time is divided into *N* intervals. The artificial spins are then represented by a piecewise constant function, where each interval consists of 20 samples. To prevent transient effects during the sample generation and the signal detection, only the last value of the 20 samples is used. In the processing stage, the signal is then processed to obtain the spin amplitude *x*_*n*_[*k*] by digitally subtracting the DC component, which is separately measured before each experiment from the light intensity passing through the modulator without any feedback signal. To calculate *f*_*n*_[*k*], a matrix multiplication is performed on the spin amplitude *x*_*n*_[*k*] according to Eq. (3). Due to the low intrinsic noise of the system, additional Gaussian white noise *ζ*_*n*_ with a standard deviation of *σ*_*ζ*_ = 0.04 is digitally injected. This could likewise be achieved by driving the system closer to the laser threshold or by adding electronic noise.

### Analogy between OEO-based and DOPO-based CIMs

To understand the analogy between DOPO-based and OEO-based CIMs, we are considering the equations of motion for a DOPO-based CIM^21^:

4 $$\frac{{{\mathrm{d}}c_n}}{{{\mathrm{d}}t}} = \left( { - 1 + p - c_n^2 - s_n^2} \right)c_n + \frac{1}{{A_s}}\sqrt {c_n^2 + s_n^2 + \frac{1}{2}} \frac{{{\mathrm{d}}\zeta _n}}{{{\mathrm{d}}t}},$$

5 $$\frac{{{\mathrm{d}}s_n}}{{{\mathrm{d}}t}} = \left( { - 1 - p - c_n^2 - s_n^2} \right)s_n + \frac{1}{{A_s}}\sqrt {c_n^2 + s_n^2 + \frac{1}{2}} \frac{{{\mathrm{d}}\zeta _{n\prime }}}{{{\mathrm{d}}t}}.$$

The equations of motion describe the temporal evolution of the in-phase component *c*_*n*_ and the quadrature component *s*_*n*_ of the optical field of the *n*th DOPO for a network of *N* DOPO pulses that circulate in a ring cavity. *p* refers to the pump parameter, where *p* = 1 represents the threshold of the pitchfork bifurcation and is thus analogous to the feedback strength *α* in the OEO-based CIM. *A*_s_ is the saturation amplitude and $\frac{{{\mathrm{d}}\zeta }}{{{\mathrm{d}}t}}$ is a Gaussian white noise term. The Ising model is implemented by coherently coupling the DOPO pulses. In measurement-feedback based CIMs, this is achieved by measuring the in-phase component $\tilde c_n[k]$ of each DOPO pulse during the roundtrip *k* with balanced homodyne detection and then using this signal to generate an electronic feedback signal $\tilde f_n$ that is injected back into the cavity by modulating a train of optical pulses^11,17^. The homodyne signal $\tilde c_n$ corresponds to the coherent superposition

6 $$\tilde c_n[k] \propto c_n[k]\,{\mathrm{cos}}\,\left( {{\mathrm{\Delta }}\varphi _n[k]} \right) + \beta \mathop {\sum}\limits_m {J_{mn}} c_m[k]\,{\mathrm{cos}}\,\left( {\Delta \varphi _m[k]} \right),$$

where Δ*φ*_*n*_ is the phase difference between the DOPOs and the local oscillator and *β* is the coupling strength. For DOPOs, the phase difference is degenerate and can only be Δ*φ* = {0, *π*}. All phase terms in the coherent superposition thus reduce to cos(Δ*φ*) = ±1, so that the electrical field of the DOPOs is real valued with a positive or negative amplitude. This allows to calculate the coherent superposition inside a FPGA, which generates the feedback signal $\tilde f_n$:

7 $$\tilde f_n[k + 1] = \mathop {\sum}\limits_m {J_{mn}} \tilde c_m[k].$$

After performing one roundtrip in the cavity, this signal is injected back into the cavity to implement the coupling. In the equations, this corresponds to adding the feedback signal $\tilde f_n[k]$ to the in-phase component after each roundtrip. During the roundtrip, the time evolution of the DOPOs is given by Eqs. (4) and (5). It is important to mention here that although their general operating principle is different, DOPO-based CIMs with purely optical coupling can be described by the same set of equations^21^.

It has been shown that arbitrary Ising Hamiltonians can be mapped to the dynamics of Eqs (4)–(6)^21^. In the following, we show that the equations of motion of DOPO-based CIMs and OEO-based CIMs become analogous when the CIM is operating close to the threshold, so that the Ising model can be mapped to the OEO-based CIM in the same way as for DOPO-based CIMs. As a first step, we further simplify the equations of motion for the DOPO-based CIM. In Eqs. (4) and (5), the noise term is multiplicative, i.e. its amplitude depends on *c*_*n*_ and *s*_*n*_. Since the noise amplitude is small compared to the spin amplitude above threshold, variations in the noise amplitude become neglectable so that we use a noise term $\frac{{\mathrm{{d}}\tilde \zeta _n}}{{{\mathrm{{d}}}t}}$ with a constant amplitude instead. Also above threshold, it is safe to neglect the quadrature component *s*_*n*_. This is reasonable since the feedback signal has no complex component and since Eq. (5) undergoes no bifurcation at *p* = 1, so that *s*_*n*_ always remains below threshold and thus $c_n \gg s_n$. This leads to the following simplified equation of motion for the in-phase component, which is equivalent to the normal form of a pitchfork bifurcation at *p* = 1 with the two stable fixed points $c_{2,3}^ \ast = \pm \sqrt {p - 1}$:

8 $$\frac{{{\mathrm{{d}}}c_n}}{{{\mathrm{{d}}}t}} = (p - 1)c_n - c_n^3 + \frac{{\mathrm{{d}}\tilde \zeta _n}}{{{\mathrm{{d}}}t}}.$$

This continuous differential method can be transformed into a discrete map by approximating it with the Euler method. For a single Euler step *c*_*n*_[*k* + 1] = *c*_*n*_[*k*] + *hf*(*t*_*n*_, *c*_*n*_(*t*_*n*_)) with step size *h*, we assume a large time step of *h* = 1 that corresponds to performing one entire cavity roundtrip. This assumes that *c*_*n*_ only changes slowly during each roundtrip, which is true for DOPOs close to the threshold^17,32^. Adding the feedback signal after each roundtrip results in the following iterative map:

9 $$c_n[k + 1] = pc_n[k] - c_n^3[k] + \beta \mathop {\sum}\limits_m {J_{mn}} \tilde c_m[k] + \tilde \zeta _n.$$

Using the assumption that the spin amplitude *x*_*n*_ is small close to the threshold, we now demonstrate that the equation of motion for OEO-based CIMs can be reduced to the same pitchfork normal form. Focusing solely on the behavior close to the threshold (*α* = *p* ≈ 1) is a reasonable assumption, since it is well known that CIMs and other gain-dissipative systems achieve the highest performance close to the threshold^13^. We can thus expand the cos^2^ in Eq. (2) as a Taylor series around *x*_*n*_ = 0 to the third order. This results in the following expression:

10 $$x_n[k + 1] = \alpha x_n[k] + \beta \mathop {\sum}\limits_m {J_{mn}} x_m[k] + \zeta _n - \frac{2}{3}\left( {\alpha x_n[k] + \beta \mathop {\sum}\limits_m {J_{mn}} x_m[k] + \zeta _n} \right)^3.$$

We are assuming that any terms related to self-feedback are in general larger than any terms related to mutual coupling, so that $\alpha x_n[k] \gg \beta \mathop {\sum}\nolimits_m {J_{mn}} x_m[k]$. While this may not be true for all cases, we will see in the following that it still presents a reasonable approximation close to the threshold. Using this assumption, we neglect the third-order terms in the feedback strength. Similarly, any higher orders terms for the noise are disregarded, which leaves us with the following relation:

11 $$x_n[k + 1] = \alpha x_n[k] - \frac{2}{3}\alpha ^3x_n[k]^3 + \beta \mathop {\sum}\limits_m {J_{mn}} x_m[k] + \zeta _n.$$

**Fig. 6:**
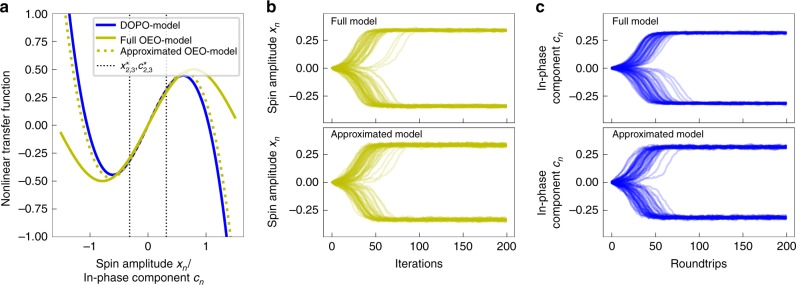
Comparison of dynamics of DOPO-based and OEO-based CIM. **a** Nonlinear transfer functions for the DOPO-model (blue line), the full OEO model (solid yellow line), and the approximated OEO model (dashed yellow line) at *α* = *p* = 1.1. The fixed points of the models are indicated by the black dashed line. **b**, **c** Simulation of the dynamics of 100 uncoupled oscillators for the OEO model (**b**) and the DOPO-model (**c**) at *α* = *p* = 1.1. The upper panels show the simulations using the full model, while the lower panels show the results of the approximated models

We find that this corresponds to the approximated DOPO model in Eq. (9). Particularly, the coupling term in the OEO model is the same as the coherent superposition of DOPO pulses in Eq. (6), showing that the output of the MZM approximates the homodyne signal for small spin amplitudes. Similar to the simplified equation of motion for the DOPO-based CIM, the simplified OEO model exhibits the same pitchfork at *α* = 1 with the stable fixed points $x_{2,3}^ \ast = \pm \sqrt {3/2(\alpha - 1)}$. Although the expression for the nonlinear transfer function contains an additional factor in the third-order term, we find that it still presents a good approximation close to the threshold. In Fig. 6a, we compare the nonlinear transfer functions for the OEO-based CIM (Eqs. (2) and (11)) and the DOPO-based CIM (Eq. (9)) close to the threshold at *α* = *p* = 1.1 and *β* = 0 and find that all expressions agree well for all values of the spin amplitude between the fixed points. We also assess the similarities between both models by comparing the time evolution of the spin amplitude *x*_*n*_ and the in-phase component *c*_*n*_ close to the threshold. In Fig. 6b, c, we show the time evolution for a network of 100 uncoupled artificial spins in the OEO-based CIM and in the DOPO-based CIM at *α* = *p* = 1.1 and *β* = 0. In the upper panel, we show the results from the simulations using the full model while the lower panel contains the results of the simplified Eqs. (9) and (11). For simulation of the full DOPO-model, we use the same set of parameters as in ref. ^17^. Comparing the full models with the approximated models, we find that both the OEO models and the DOPO models agree very well. Furthermore, when comparing the dynamics of the OEO-based CIM to the DOPO-based CIM, we find that they are almost indistinguishable. In all models, we can observe the spin states bifurcating to the same fixed points at very similar timescales. Due to the demonstrated analogy of the equations of motion, it can thus be shown that an OEO-based CIM operates analogous to a DOPO-based CIM, which is for example also reflected in the domain dynamics that we observe for the 2D Ising model in Fig. 3. An OEO-based CIM thus possesses the same capability to implement arbitrary Ising Hamiltonians as its DOPO-based counterpart, which is also supported by our benchmarks.

### Monte Carlo simulations

The Monte Carlo simulations were performed using the Metropolis–Hastings algorithm, which is a local update method. For each iteration, a single spin is flipped and the change in energy Δ*E* is considered. If the energy decreases, the spin remains flipped. If the energy increases, the spin remains flipped with a probability of

12 $$P = 1 - {\mathrm{exp}}( - {\mathrm{\Delta }}E/k_{\mathrm{B}}T).$$

The spins are flipped sequentially in a typewriter ordering, which is known to be effective for 2D graphs. We also checked the success rate to reach the ground state with a random update order and found that the method performs worse in terms of success rate and calculation time. To implement a Hopfield network, we have set the temperature to *T* = 0.01. At this low temperature, the update mechanism only flips a spin if the overall energy decreases, which is identical to the deterministic update rule of Hopfield networks. To reach the lowest energy state, 300,000 iterations were performed for each simulation.

To verify the ground states found by the CIM, we have also implemented simulated annealing by gradually decreasing the temperature of the Metropolis–Hastings algorithm at each iteration *k*. We have applied an exponential temperature schedule *T*[*k*] = 2 exp(−0.0001 *k*) and simulated the spin network until the energy remained unchanged. For long annealing times, it is known that simulated annealing can converge to the global minimum.

## Data Availability

The authors declare that all relevant data are included in the manuscript. Additional data are available from the corresponding author upon reasonable request.
